# Supplementary material for: DNA damage causes rapid accumulation of phosphoinositides for ATR signaling
Source: Nat Commun. 2017 Dec 14;8:2118. doi: 10.1038/s41467-017-01805-9 (PMC5730617; doi:10.1038/s41467-017-01805-9)
Supplement: Supplementary file 1 — Supplementary Information [file 41467_2017_1805_MOESM1_ESM.pdf]

Supplementary Figure 1

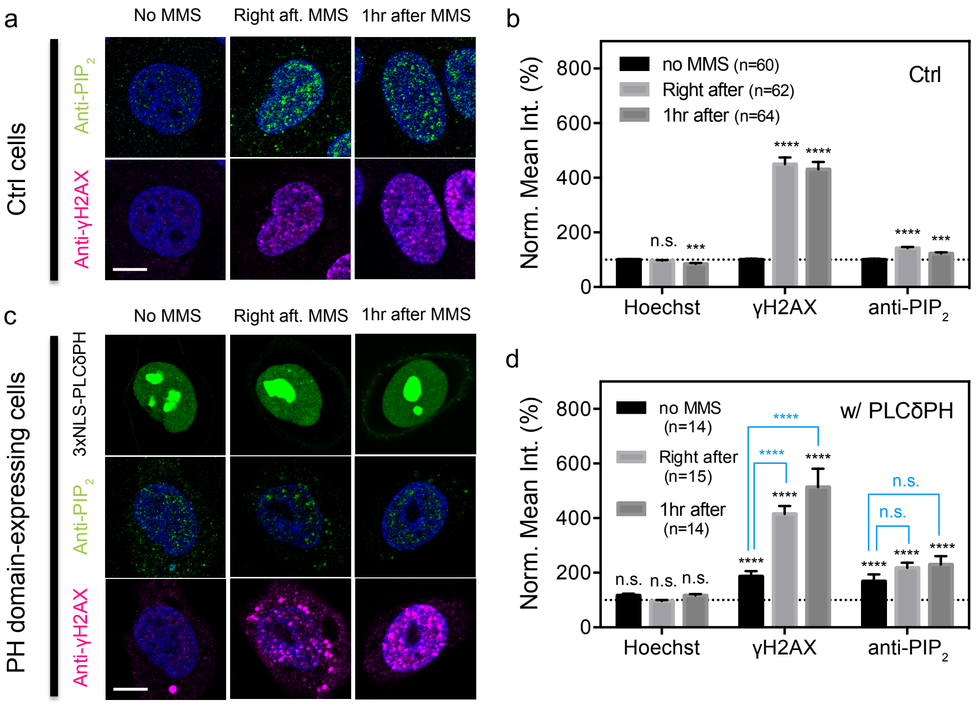

Supplementary Figure 1. **Nuclear PIP_2/PIP_3 level increases after chemically induced DNA damage.** (a) Confocal optical slices showing nuclear PIP_2 speckles and γH2AX foci in control U2OS cells as revealed by immunostaining before and after being exposed to 0.01% MMS for 2 hours. Secondary antibody labels for PIP_2 and γH2AX were conjugated with Alexa Fluor-546 and Alex Fluor-647, respectively. (b) Corresponding quantification of panel A showing the normalized mean fluorescence intensity of Hoechst 33342, PIP_2 and γH2AX within the nucleus. (c) Same anti-PIP_2 and anti-γH2AX staining for U2OS cells transfected with 3xNLS-PLCδPH before and after adding 0.01% MMS for 2 hours. (d) Corresponding quantification of panel c. Results were normalized to the signal intensities of non-UV treated, non-transfected cells as shown in panel b. Symbols in black indicated t-test p-values when comparing it to the signal intensity of non-UV treated, non-transfected cells in panel b. Symbols in blue indicated t-test results when comparing it to the non-UV treated PLCδPH-expressing cells. Error bars represent mean±s.e.m. Data are representative of three independent experiments. Statistical significance was determined by Student's t-test. *$p<0.05$; ***$p<0.001$; ****$p<0.0001$. n.s. represents not significant. Scale bar 10 μm. (See also Figure 1 for comparison)

Supplementary Figure 2

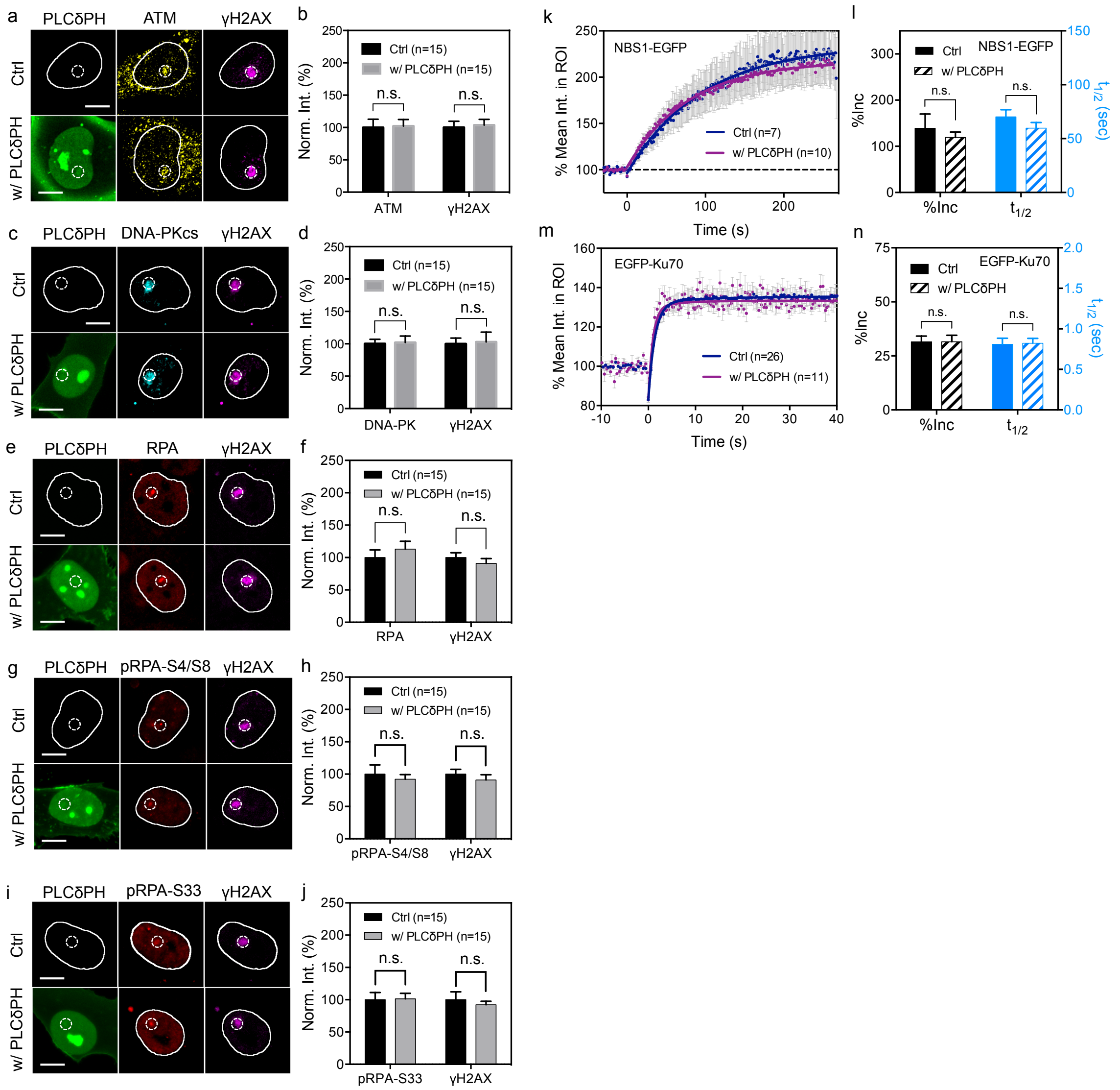

Supplementary Figure 2. **PH domain in the nucleus does not suppress recruitment of ATM, DNA-PKcs and RPA upon laser microirradiation.** (a) Representative micrographs of control and PLCδ-PH-expressing cells stained with ATM and γH2AX after laser microirradiation and (b) the corresponding quantification of normalized mean fluorescence intensity within the ROI. Similar experiments and analyses were performed for other proteins including (c&d) DNA-PKcs; (e&f) RPA; (g&h) pRPA-S4/S9; (i&j) pRPA-S33. (k) The recruiting dynamics of NBS1-EGFP in the presence and absence of 3xNLS-PLCδ-PH-mCherry upon laser microirradiation and (l) the corresponding %Inc and half time from a single exponential fitting. (m) The recruiting dynamics of EGFP-Ku70 in the presence and absence of 3xNLS-PLCδPH-mCherry upon laser microirradiation and (n) the corresponding %Inc and half time from a single exponential fitting. Dashed circles indicate the ROI for laser microirradiation. Data are representative of three independent experiments. Error bars represent mean±s.e.m. Student's t-test, n.s. represents not significant. Scale bar 10 μm.

Supplementary Figure 3

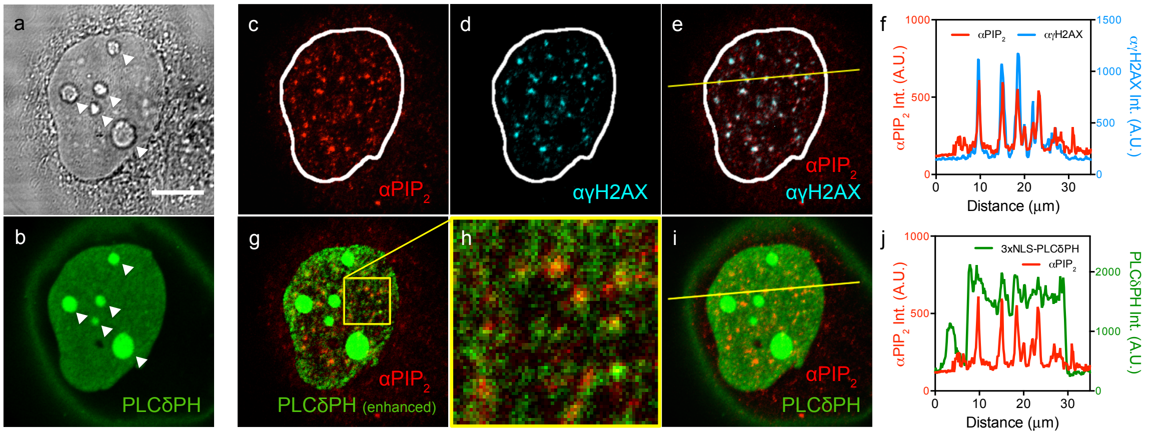

Supplementary Figure 3. **Co-localization of anti-PIP_2 speckes, γH2AX foci and local enrichment of PLCδ-PH domain in UV-damaged cells.** (a) Bright field and (b) fluorescent micrographs of U2OS expressing NLS-tagged PLCδPH-EGFP. White arrowheads indicate the nucleoli. The enrichment of NLS-tagged construct in the nucleoli is a typical artifact from the NLS tag, but not the PH domain. The cell was fixed and stained for (c) anti-PIP_2 and (d) anti-γH2AX antibody after being exposed to UV for 2 min followed by one-hour recovery in an incubator. (e) Overlay of anti-PIP_2 and anti-γH2AX staining revealed co-localization of PIP_2-enriched speckles and γH2AX foci. (f) Corresponding intensity line profile along the yellow line indicated in panel E. (g) Enhanced fluorescent micrograph of 3xNLS-PLCδPH overlaid with anti-PIP_2 staining. (h) Zoom-in view of panel H revealed the enrichment of PLCδPH around anti-PIP_2 speckles. (i) Anti-PIP_2 staining overlaid with NLS-tagged PLCδPH-EGFP without contrast enhancement. (j) Corresponding intensity line profile along the yellow line indicated in panel I showed discernable co-localization of anti-PIP_2 speckles with the fine structure of PLCδPH domain in the nucleus. Images are representative of three independent experiments. Scale bar 10 μm.

Supplementary Figure 4

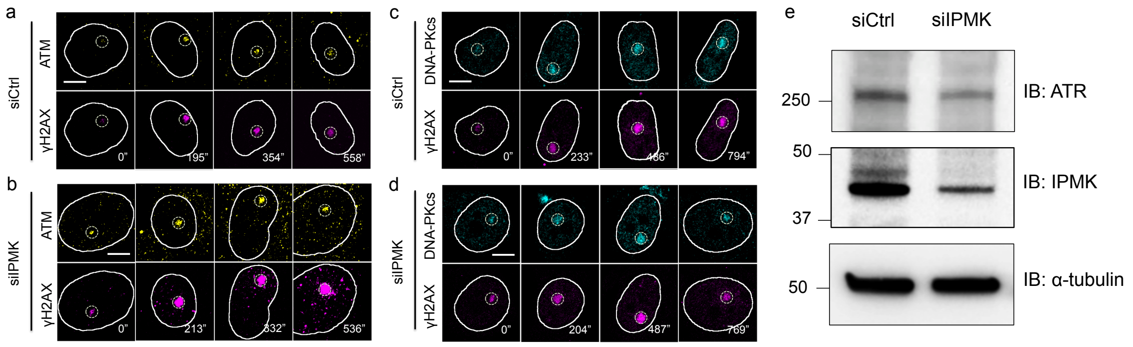

Supplementary Figure 4. **IPMK depletion promotes ATM accumulation but has no affect on DNA-PKcs recruitment.** (a&b) ATM and γH2AX immunofluorescent micrographs of U2OS nucleus upon laser microirradiation over time at control and IPMK-depleted conditions, respectively. (c&d) Same experiments stained for DNA-PKcs and γH2AX upon laser microirradiation over time at control and IPMK-depleted conditions, respectively. Refer to Figure 4o for data quantification. (e) ATR expression level was not affected by transient depletion of IPMK after 72 hrs. Images are representative of three independent experiments.

Supplementary Figure 5

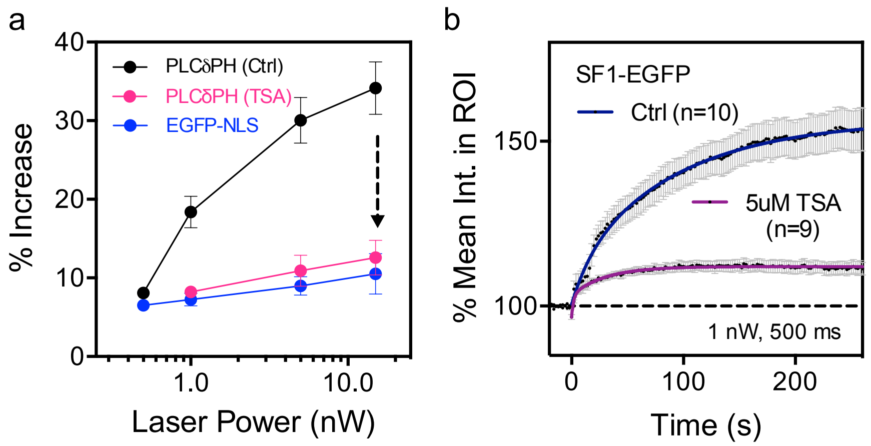

Supplementary Figure 5. **HDAC inhibitor suppresses PLCδPH and SF1 accumulation at damage sites.** (a) The accumulation magnitude of nuclear PLCδPH was nearly abolished to a background level, as indicated by EGFP-3xNLS at the presence of 5 μM Trichostatin A (TSA). (b) The accumulation magnitude of EGFP-fusion SF1 was also greatly suppressed by the presence of TSA at the same concentration. Data are representative of three independent experiments. Error bars represent mean±s.e.m.

Supplementary Figure 6

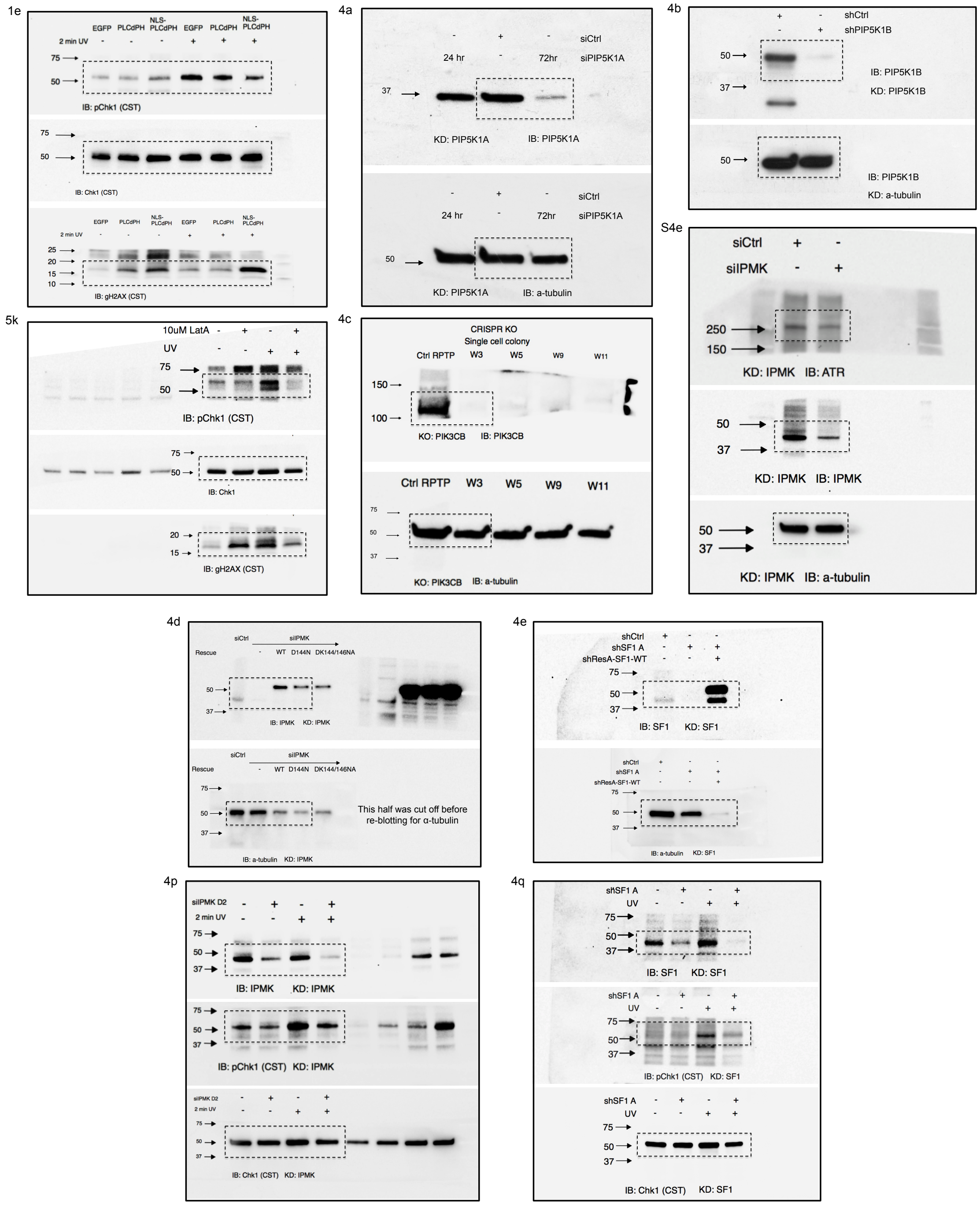

Supplementary Figure 6. **Uncropped western blot images of corresponding figures presented.** Dashed box indicates regions of interest. Numbers on the left indicated molecular weight (kDa) of the ladders. IB: immunoblotting; KD/KO: knock down/knock out. All blots are representative of at least three independent experiments.

Supplementary Table 1- **List of cloning and sequencing primers**

Primers for cloning by restriction enzyme				
Template	Action	Final Construct	Primer	Remark
BtkPH-EGFP	Add 3xNLS	3xNLS-BtkPH-EGFP	5'-gagtCTCGAGCatgGATCCAAAAAAGAAGAGAAAGGTAG-3'	XhoI
			5'-gagtGAATTCAGATCCGGTGGATCCTACCTT-3'	EcoRI
PLCdPH-EGFP	Add 3xNLS	3xNLS-PLCdPH-EGFP	5'-gagtCTCGAGatgGATCCAAAAAAGAAGAGAAAGGTAG-3'	XhoI
			5'-gagtGAATTCGAGATCCGGTGGATCCTACCTT-3'	EcoRI
EGFP-P4M-SidMx2	Add 3xNLS	3xNLS-EGFP-P4M-SidMx2	5'-gagtGCTAGCGATGGATCCAAAAAAGAAGAGAAAGGTAG-3'	NheI
			5'-gagtACCGGTAGAGATCCGGTGGATCCTACCTT-3'	AgeI
mCherry-P4M-SidM	Add 3xNLS	3xNLS-mCherry-P4M-SidM	5'-gagtGCTAGCGATGGATCCAAAAAAGAAGAGAAAGGTAG-3'	NheI
			5'-gagtACCGGTAGAGATCCGGTGGATCCTACCTT-3'	AgeI
3xNLS-PLCdPH-EGFP	Replace EGFP with mCherry	3xNLS-PLCdPH-mCherry	5'-gactACCGGTCGCCACCATGGTGAGCAAGG-3'	AgeI
			5'-CTACTTGTACAGCTCGTCCATGCC-3'	BsrGI
3xNLS-BtkPH-EGFP	Replace EGFP with mCherry	3xNLS-BtkPH-mCherry	5'-gactACCGGTCGCCACCATGGTGAGCAAGG-3'	AgeI
			5'-CTACTTGTACAGCTCGTCCATGCC-3'	BsrGI
NBS1-turboGFP	Replace turboGFP with EGFP	NBS1-EGFP	5'-gactgctagcATGTGGAAACTGCTGCCCGC-3'	NheI
			5'-gactCTCGAGCGGCCGCGTACGCG-3'	XhoI
SF1-turboGFP	Replace turboGFP with EGFP	SF1-EGFP	5'-cagtAGATCTGCCGCCGCGATCGCCATG-3'	BglII
			5'-cagtCTCGAGCGGCCGCGTACGCG-3'	XhoI
3xNLS-BtkPH-EGFP	Move EGFP from C-term to N-term	EGFP-3xNLS-BtkPH	5'-gactAGATCTATGGATCCAAAAAAGAAGAGAAAG-3'	BglII
			5'-gactGTCGACttaGGTGGCGACCGGTGGATCCG-3'	SalI
3xNLS-BtkPH(R28C)-EGFP	Move EGFP from C-term to N-term	EGFP-3xNLS-BtkPH-R28C	5'-gactAGATCTATGGATCCAAAAAAGAAGAGAAAG-3'	BglII
			5'-gactGTCGACttaGGTGGCGACCGGTGGATCCG-3'	SalI
3xNLS-PLCdPH-EGFP	Move EGFP from C-term to N-term	EGFP-3xNLS-PLCdPH	5'-gactAGATCTATGGATCCAAAAAAGAAGAGAAAG-3'	BglII
			5'-gactGTCGACttaGGTGGCGACCGGTGGATCCT-3'	SalI
3xNLS-PLCdPH(R40L)-EGFP	Move EGFP from C-term to N-term	EGFP-3xNLS-PLCdPH-R40L	5'-gactAGATCTATGGATCCAAAAAAGAAGAGAAAG-3'	BglII
			5'-gactGTCGACttaGGTGGCGACCGGTGGATCCT-3'	SalI
3xNLS-PLCdPH(K30L-K32L-R40L)-EGFP	Move EGFP from C-term to N-term	EGFP-3xNLS-PLCdPH-K30L-K32L-R40L	5'-gactAGATCTATGGATCCAAAAAAGAAGAGAAAG-3'	BglII
			5'-gactGTCGACttaGGTGGCGACCGGTGGATCCT-3'	SalI

Primers for cloning by site-directed mutagenesis				
Template	Action	Final Construct	Primer	Remark
3xNLS-PLCdPH-mCherry	Remove 3xNLS	PLCdPH-mCherry	5'-TCAAGCTTCGAATTCACGGCATG-3'	Designed by NEBaseChanger online tool
			5'-GCTCGAGATCTGAGTCCGGTAG-3'	
3xNLS-PLCdPH-R40L-mCherry	Remove 3xNLS	PLCdPH-R40L-mCherry	5'-TCAAGCTTCGAATTCACGGCATG-3'	
			5'-GCTCGAGATCTGAGTCCGGTAG-3'	
3xNLS-PLCdPH-K30L-K32L-R40L-mCherry	Remove 3xNLS	PLCdPH-K30L-K32L-R40L-mCherry	5'-TCAAGCTTCGAATTCACGGCATG-3'	
			5'-GCTCGAGATCTGAGTCCGGTAG-3'	
3xNLS-BtkPH-mCherry	Remove 3xNLS	BtkPH-mCherry	5'-TCAAGCTTCGAATTCCAGAAAGAAGAAG-3'	
			5'-GCTCGAGATCTGAGTCCGGTAG-3'	

3xNLS-BtkPH-R28C-mCherry	Remove 3xNLS	BtkPH-R28C-mCherry	5’-TCAAGCTTCGAATTCCAGAAAGAAGAAG-3’	
			5’-GCTCGAGATCTGAGTCCGGTAG-3’	
3xNLS-BtkPH-EGFP	Introduce R28C point mutation	3xNLS-BtkPH(R28C)-EGFP	5'-TAAACTTCAAGAAGTGCCTGTTTCTCTTGA-3'	
			5'-TCAAGAGAAACAGGCACTTCTTGAAGTTTA-3'	
3xNLS-PLCdPH-EGFP	Introduce R40L point mutation	3xNLS-PLCdPH(R40L)-EGFP	5'-ATGGAGGAGAGAGCTGTTCTACAAGTTGCA-3'	
			5'-TGCAACTTGTAGAACAGCTCTCTCCTCCAT-3'	
3xNLS-PLCdPH(R40L)-EGFP	Introduce K30L and K32L point mutation	3xNLS-PLCdPH(K30L-K32L-R40L)-EGFP	5'-CCAGCTCCTGCTGGTGCTGTCCAGC-3'	
			5'-CTGCCCTTCAGCAGCGCC-3'	
3xNLS-BtkPH-mCherry	Introduce R28C point mutation	3xNLS-BtkPH(R28C)-mCherry	5'-TAAACTTCAAGAAGTGCCTGTTTCTCTTGA-3'	
			5'-TCAAGAGAAACAGGCACTTCTTGAAGTTTA-3'	
3xNLS-PLCdPH-mCherry	Introduce R40L point mutation	3xNLS-PLCdPH(R40L)-mCherry	5'-ATGGAGGAGAGAGCTGTTCTACAAGTTGCA-3'	
			5'-TGCAACTTGTAGAACAGCTCTCTCCTCCAT-3'	
3xNLS-mCherry-P4M-SidM	Introduce K23A point mutation	3xNLS-mCherry-P4M-SidM (K23A)	5'-TGATGCTTTAGCAACAGAAATCCTGGC-3'	
			5'-CCTCGCATTTGCTGATATTTTTC-3'	
mSF1-WT-Myc-DDK	silent mutation shRNA-resistant	shResA-mSF1-WT-Myc-DDK	5'-CAATAAAGTCGGAATATCCAGAGCCCTATGCC-3'	
			5'-TTCGATTCGAAAATGCAGGATAGAGGTAGCCA-3'	
**Primers for sequencing**				
Usage			Primer	Remark
Sequencing primer for pRFP-C-RS shRNA constructs			5’-TTGAGATGCATGCTTTGCATAC-3’	Reverse
Sequencing primer for turboGFP constructs			5’-GGACTTTCCAAAATGTCG-3’	Forward
Sequencing primer for turboGFP constructs			5’-ATTAGGACAAGGCTGGTGGG-3’	Reverse
