# Supplementary material for: DNA damage causes rapid accumulation of phosphoinositides for ATR signaling
Source: Nat Commun. 2017 Dec 14;8:2118. doi: 10.1038/s41467-017-01805-9 (PMC5730617; doi:10.1038/s41467-017-01805-9)
Supplement: Supplementary file 3 — Description of Additional Supplementary Files [file 41467_2017_1805_MOESM3_ESM.pdf]

Description of Additional Supplementary Files

File Name: Supplementary Movie 1
Description: **Rapid accumulation of nuclear PLCδPH domain at a damage site.** A mouse embryonic fibroblast expressing 3xNLS-tagged PLCδPH-EGFP was microirradiated with a 355 nm UV laser and showed a rapid accumulation of PH domain at the damage site. The same probe also bound to the nucleoli, which was a known feature of proteins tagged with NLS derived from SV40 large T-antigen. The laser was introduced at time zero at 15 nW, as measured from the back aperture of the objective, for 500 ms. Movie was captured by an A1R confocal with a 60x/NA1.4 oil objective (Nikon) under resonant scanning mode with a frame rate at 3.75 frame per second. Scale bar, 10 µm.
